# No lukewarm diatom communities—the response of freshwater benthic diatoms to phosphorus in streams as basis for a new phosphorus diatom index (PDI_SE_)

**DOI:** 10.1007/s10661-023-11378-4

**Published:** 2023-06-06

**Authors:** Maria Kahlert, Jens Fölster, Kálmán Tapolczai

**Affiliations:** 1 Department of Aquatic Sciences and Assessment, Swedish University of Agricultural Sciences, PO Box 7050, 750 07 Uppsala, Sweden; 2 Balaton Limnological Research Institute, Eötvös Loránd Research Network (ELKH), Klebelsberg Kuno u. 3, Tihany, Hungary

**Keywords:** Water Framework Directive, Bioassessment, Community, Eutrophication, Quality index, TDI

## Abstract

In the present study, we developed a new Swedish phosphorus diatom index (PDI_SE_) to improve the poor fit of existing indices to match the needs of water managers to detect and mitigate eutrophication. We took advantage of a large amount of data (820 Swedish stream sites) collected in recent years. During our work, we found an unexpected bimodal response of the diatom assemblages to phosphorus. The taxa clustered either into an assemblage with a low or with a high site-specific averaged TP optimum (a calculated value comprised of the diatom taxa-specific optima). We could not find a characteristic diatom assemblage for sites with intermediate site-specific averaged TP optima. To our knowledge, this bimodal community response has not been shown earlier. The PDI_SE_ correlated more strongly than the currently used TDI to changes in TP concentrations. Thus, the PDI_SE_ should replace the TDI in the Swedish standard method. The modeled TP optima (expressed as categories) were different compared to the TDI for most of the taxa included in the index, indicating that the realized niche for these morphotaxa was different between Sweden and the UK where the TDI was developed originally. With a *r*^2^ of 0.68, the correlation of the PDI_SE_ to TP is among the highest reported for other diatom nutrient indices globally; thus, we believe that it might be worth to test it for other bioregions with similar geography and climate.

**Supplementary Information:**

The online version contains supplementary material available at 10.1007/s10661-023-11378-4.

## Introduction

Freshwater benthic diatoms are known as good indicators for local water chemistry (Smol & Stoermer, 2010), and are therefore widely used as part of standard bioassessment toolkits (Charles et al., 2021), for example, for monitoring of water bodies in accordance with the United States Clean Water Act (1972) or the European Water Framework Directive (WFD: European Parliament & Council of the European Union, 2000). One important field of usage is the detection of eutrophication and the follow-up of countermeasures against it. Eutrophication is still considered the most common stressor in freshwaters, and many different indicators have been developed and implemented to detect increased nutrient concentrations with the help of changes in the benthic diatom flora (Carvalho et al., 2019; European Environment Agency, 2018; Poikane et al., 2021). After almost two decades of using these methods intensively, a vast amount of data are now available to better analyze pressure-response relationships than possible at the time when the methods were first implemented. Those data can now also be used to examine if established class boundaries for water quality might need adjustment as discussed by Poikane et al. (2021).

An example of a diatom nutrient index in need of possible adjustment is the use of the TDI (Trophic Diatom Index, Kelly, 1998; Kelly & Whitton, 1995) in Sweden. The TDI is an index originally developed in the UK, based on filterable reactive phosphorus (P) optima and tolerances of littoral diatoms working well as trophy indicator for UK sites without organic pollution (Kelly & Whitton, 1995). Kelly and Whitton (1995) assigned each taxon a “sensitivity value” based upon observed P optimum, and an “indicator value” based on the tolerance. The TDI was chosen during the WFD implementation process because Sweden decided to rather introduce a robust existing index instead of developing a Swedish index from scratch without sufficient own data. The TDI was relatively widely accepted not only in the UK but also in other countries. It was one of the intercalibrated indices being part of the implementation process for the WFD (Kelly et al., 2009, 2014), and it was used already in Finland, Sweden’s neighboring country with a similar diatom flora (Eloranta & Soininen, 2002). Sensitivity and indicator values of taxa can depend strongly on local geochemical conditions (Rott & Schneider, 2014; Rott et al., 2003). Thus, it is always important to test if any index is suitable at all if used in a new biogeographical region. The results for the TDI on the few existing data from Sweden confirmed that the TDI gave an acceptable response to phosphorus, the main nutrient responsible for eutrophication in Swedish freshwaters (Fölster et al., 2021), and also the Finnish class boundaries for ecological status seemed to be suitable for Swedish conditions. While the main national standard index to classify ecological status for the WFD is the IPS (Indice de Polluo-Sensibilité Spécifique; Cemagref, 1982), the TDI is used as supporting index to show if an indicated bad status is due to nutrient or to other factors such as organic pollution (Havs- och vattenmyndigheten, 2018a). Whereas the IPS as main index had been carefully analyzed for pressure-response relationships and adjusted to fit Swedish conditions (e.g., adding many species-specific sensitivity values for Nordic species lacking in the original version of the IPS (Kahlert et al., 2018), the TDI was used with some minor changes only (e.g., Lindegarth et al., 2016).

However, with more data becoming available, it became clear that the TDI, as used in Sweden, can indicate the nutrient impact only grossly (Kahlert, 2011). Furthermore, an updated version of the TDI (Kelly et al., 2008) was not indicating nutrient concentrations in Sweden in a satisfying way (unpublished data). Additionally, water administration boards requested an improved diatom nutrient indicator with less uncertainty to be used to detect and mitigate eutrophication (Havs- och Vattenmyndigheten, 2018b). The water administration boards requested additionally a better linkage between the recently updated chemical targets for environmental assessment with biological responses (Fölster et al., 2021). Last but not least, there was a gap of knowledge about how individual taxa aggregate to diatom assemblages in response to a phosphorus gradient, especially for Nordic regions. Whereas the development of taxon-specific index values as basis for nutrient indices has been a field of research since a long time in both lakes and streams (Bradshaw & Anderson, 2001; Schönfelder et al., 2002; Smol & Stoermer, 2010), the pattern of change of the aggregated taxon composition has received much less attention, especially in streams.

Analyzing the benthic diatom assemblages and chemical data of 820 Swedish stream sites, our objectives in the present study were to (i) overhaul the performance of the TDI in terms of its correlation to the total phosphorus (TP) gradient and taxon-specific TP optima; (ii) perform a structured development of a national diatom index (phosphorus diatom index; PDI_SE_) dedicated to the reflection of phosphorus, and assess its performance compared to the TDI and IPS indices; and to (iii) study the structure of diatom communities along the TP gradient.

## Material and methods

### Data

**Fig. 1:**
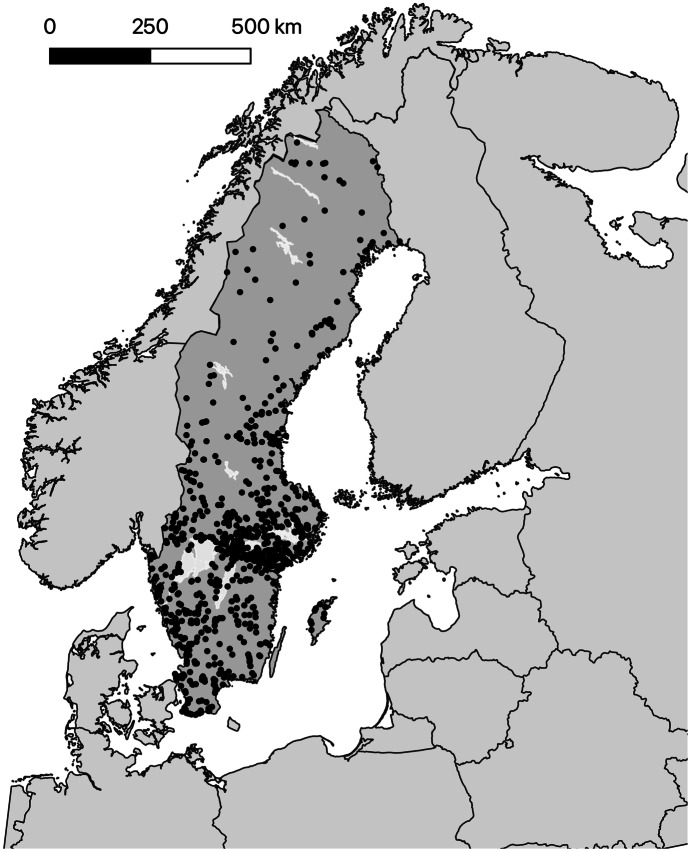
Studied Swedish stream sites with diatom and water chemistry data (extract from Miljödata-MVM July 2021, *n* = 820 sites)

We used for this study all available quality-controlled diatom data for Swedish streams, most of them collected for routine monitoring programs. Those data are stored by the national data host for lakes and watercourses in the open access database Miljödata-MVM (Miljödata-MVM, 2022). This database includes biological as well as chemical data from national, regional, and recipient freshwater monitoring on mission of the Swedish Agency for Marine and Water Management. We downloaded data for all available stream sites with both diatom and water chemistry, summing up to 820 sites being collected at least once for both (Fig. 1). Data covered the period 2006–2019. If more than 1 year’s data were available for a site, we chose the year 2016 where most samples were available, or the year closest to it, for further analysis.

The stream sites were distributed throughout Sweden (Fig. 1), most of them situated in Southern Sweden (ecoregion central plains, 634 sites), but also covering the other Swedish ecoregions (Northern Sweden (boreal zone) ≤ 200 m (87 sites), N. Sweden (boreal zone) 200–800 m (95 sites), N. Sweden ≥ 800 m altitude (subalpine zone, 4 sites) (following the water management administration system (Havs- och vattenmyndigheten, 2017)). Median altitude was 63 m (range: 0–1078 m). The streams also covered almost all of the Swedish stream types, which are classified by catchment size (< 100, 100–1000, > 1000 km^2^) and stream slope (< 0.1, 0.1–2, > 2%) (Havs- och vattenmyndigheten, 2017). Median pH was 7.0 (range 4.6–8.4) and TP 25.3 (1–902) µg/l. Details of the stream characteristics can be found in Supplement Table 1.

We performed a Principal Components Analysis (PCA) of the sampling sites to confirm that the sites included in this study were well distributed along a nutrient gradient, and to show the impact of other important environmental variables (included total phosphorus (TP), phosphate (P-PO_4_^3−^), pH, nitrate (N-NO_3_^−^), and ammonium (N-NH_4_^+^), *n*: 513 sites with a full set of variables). For analysis, we used the annual mean of the water chemistry variables. Variables were log10 transformed (except for pH) to obtain normal distribution of data. Prior the PCA, data were scaled to have a mean of 0 and unit variance. In most cases, diatoms and water chemistry data were available for the same year; otherwise, we chose data with the smallest time difference. Then, we only used TP for the index development.

Diatoms had been sampled in autumn following the European standard (EN 13946:2014 CEN, 2014a). According to this standard, samples shall in principle be taken from hard natural submerged substrates, i.e., stone scrapes, if available. The standard also prescribes that at least 5 stones along a stretch of 10 m and within the main flow of the river should be sampled if possible, and then pooled to one sample. Diatom analyses followed the European standard (EN 14407:2014 CEN, 2014b), and diatom identification was harmonized. In short, the samples were oxidized with hydrogen peroxide and the cleaned diatom valves then mounted with Naphrax (Brunel Microscope Ltd) on microscope slides. Identification to the lowest taxonomic level possible was done under a light microscope with interference contrast (1000 × magnification). At least 400 intact valves per sample were counted and identified using standard literature (Havs- och vattenmyndigheten, 2018a). The Swedish standard method for using diatoms in environmental assessment includes all diatom taxa. It furthermore advises to assign the counted valves of the *Achnanthidium minutissimum* complex to one of three groups based on the mean cell width of 20 individuals of one sample: group 1 with mean width < 2.2 μm, group 2 with mean width 2.2–2.8 μm, group 3 with mean width > 2.8 μm (Kahlert et al., 2007, 2009). All counts were then expressed as relative abundance.

### Taxa harmonization

Some taxonomy harmonization work needed to be applied to the compiled diatom data, because different taxa lists had been used for taxa upload. The accepted taxonomy of the Swedish diatom taxa list for freshwater diatoms (Kahlert et al., 2018) was used for taxa names in general, and for the final names of merged synonyms, except for taxa that had been split during recent taxonomical research. The latter were merged back to the common older synonym to enable the analysis of harmonized taxa units. In most cases, the term “taxon” is referring to species level, as this is the aimed level of identification of the Swedish diatom method.

### Index development including analysis of taxa composition

We performed an analysis of the diatom taxa composition, and how it related to TP and pH, to confirm that phosphorus was a main driving variable, and to get an overview about differences in diatom composition. TP and pH are the main factors driving the Nordic diatom assemblage structure (Kahlert & Gottschalk, 2014; Kahlert et al., 2021). The structure of the total 820 diatom communities was analyzed with non-metric multidimensional scaling (NMDS) with *k* = 3 dimensions (Minchin, 1987) of the Bray–Curtis community dissimilarities on taxa’s relative abundance, using the vegan v2.6–2 R package (Oksanen et al., 2022). Additionally, vectors of TP and pH were fit on the NMDS ordination plot to study their correlation with community structure.

The new diatom index dedicated to the reflection of phosphorus was developed based on improved taxon-specific TP optima and tolerance values adapted to Swedish conditions. For this purpose, we used the full taxa database of 820 samples with associated relative abundances. Using a cross-validation method following Tapolczai et al. (2019) and Tapolczai et al. (2021), the database was split randomly so that 75% of the data (615 samples with associated taxa abundances) formed the training dataset and the remaining 25% formed the test dataset (205 samples). The above-mentioned random splitting of the dataset was repeated 100 times, which resulted in 100 training and 100 test datasets. Like that, all samples were sorted into both the training and the test dataset, several times. A filtering step was applied on the training dataset, so that only taxa occurring in at least 10 samples in the training dataset were used, the remaining taxa were removed. The purpose of this step was to ensure that the ecological profiles identified in the subsequent steps were stable and based on an adequate number of occurrences. A sloppier threshold kept more taxa but with unstable profiles, while a too strict threshold reduced the number of involved taxa leading to an unstable index; this was tested in a pilot analysis. Using an occurrence limit of 10, we identified the ecological values of 455 taxa from the total 971 taxa. We identified taxon-specific TP optima and tolerance using the training datasets, and pooled the taxa into fixed TP categories. We categorized the taxon-specific values into classes to optimize response of the index to the TP gradient. Using classes helped to obtain a more linear relationship and homogenous distribution of index values (see “Results and discussion”). To identify the taxon-specific TP optima, we calculated the weighted mean of the log10 transformed TP values in the training dataset samples, using the abundances of the given taxa as weight. Since the sample composition, and so the taxa composition, of the 100 training datasets differed, a mean ecological optimum was calculated at the end for each taxon. Optima values were then classified into categories (sensitivity classes) as follows: class 1 (0–1.1 log_10_ µg/l TP), class 2 (1.1–1.2), class 3 (1.2–1.5), class 4 (1.5–1.7), class 5 (1.7–3). Similarly, tolerance values were calculated with weighted standard deviation. These values were then categorized into classes (indicator classes) 1, 2, and 3, with equal intervals (Supplement Tab. S2).

Using the identified taxon-specific sensitivity and indicator categories, we then calculated the new Swedish phosphorus diatom index (PDI_SE_) for the samples in the test database, using the formula:

$${PDI}_{SE}=\frac{{\sum }_{j=1}^{n}{a}_{j}{s}_{j}{v}_{j}}{{\sum }_{j=1}^{n}{a}_{j}{v}_{j}},$$

where *a*_*j*_ is the square root transformed abundance of the *j*^th^ taxa at that particular site the index was calculated on, *s*_*j*_ is the sensitivity value of the *j*^th^ taxa, and *v*_*j*_ is the indicator value of the *j*^th^ taxa. Since there were 100 test datasets, as many index values were calculated to a given sample as many times it appeared in the test datasets. From this, a mean index value was calculated.

In order to test the efficiency of the developed index, the calculated values were correlated to the respective TP values. Additionally, to compare the PDI_SE_ to the current Swedish diatom standard indices, it was correlated to the main index IPS and the supporting index TDI. To understand where and how the PDI_SE_ could be used to support ecological status classification in an improved version of phosphorus indication, we used the correlation equation for IPS and the PDI_SE_ and calculated the new index values for the current boundaries of ecological status classes for the WFD environmental assessment, and the corresponding TP values using the PDI_SE_ correlation to TP. Last, we compared how those TP values correspond to earlier phosphorus values reported in the background report of implementation of the diatom method for Sweden (Kahlert et al., 2007).

Ideally, the PDI_SE_ should enable a linear indication of TP. However, even if the PDI_SE_ showed an improved response to TP compared to IPS and TDI, we still observed a somewhat bimodal response. Therefore, we studied if we could explain this response by a non-unimodal distribution of TP in our dataset, and also tested the distribution of taxon-specific TP optima and site-specific mean TP optima for unimodality. Site-specific TP optima were defined by calculating the abundance-weighted mean of taxon-specific optima based on the taxa present at the given site. Unimodality was tested with Hartigans’ dip test (Hartigan & Hartigan, 1985) and performed with the diptest v0.76–0 R package (Maechler, 2022). To understand the observed TP response, we additionally used SIMPER (similarity percentage) in PAST 4.03 (Hammer, 2001) comparing the taxa composition of the group of sites with low, high, and intermediate TP average site-specific TP optima, assessing the taxa which were primarily responsible for the observed differences between these groups of samples. We defined the three groups of samples by separating all our sites by two TP thresholds, and including all sites with TP lower than 20.1 µg/l site-specific TP optima into the low TP group, and vice versa all above the second threshold of 37.2 µg/l into the high TP group, and those between into the intermediate, “lukewarm” group of samples. In order to define these thresholds, first a density plot based on kernel density estimation (Silverman, 1981) was created from the distribution data of the weighted means of TP optima per sites, and the location of the two peaks (modes) in log_10_ µg/l TP of the bimodal distribution was defined (Fig. S1A). We hypothesized an overlying combination of two symmetric unimodal distributions. In a first step, the range between the minimum log_10_ µg/l TP value to the log_10_ µg/l TP value of the first mode was defined and the first interquartile was selected (Fig. S1B). The difference between this point and the value of the first mode was added to the value of the first mode’s value, representing the threshold between the low TP and the “lukewarm” groups (1.30 log_10_ µg/l = 20.1 µg/l; Fig. S1C). Following this method, the range between the second mode and the maximum log_10_ µg/l TP value was defined, and the third interquartile was selected (Fig. S1B). The difference between this point and the value of the second mode was subtracted from the second mode’s value, representing the threshold between the “lukewarm” and the high TP groups (1.57 log_10_ µg/l = 37.2 µg/l; Fig. S1C-D).

## Results and discussion

### Clustering patterns of diatom communities, and response to TP

**Fig. 2:**
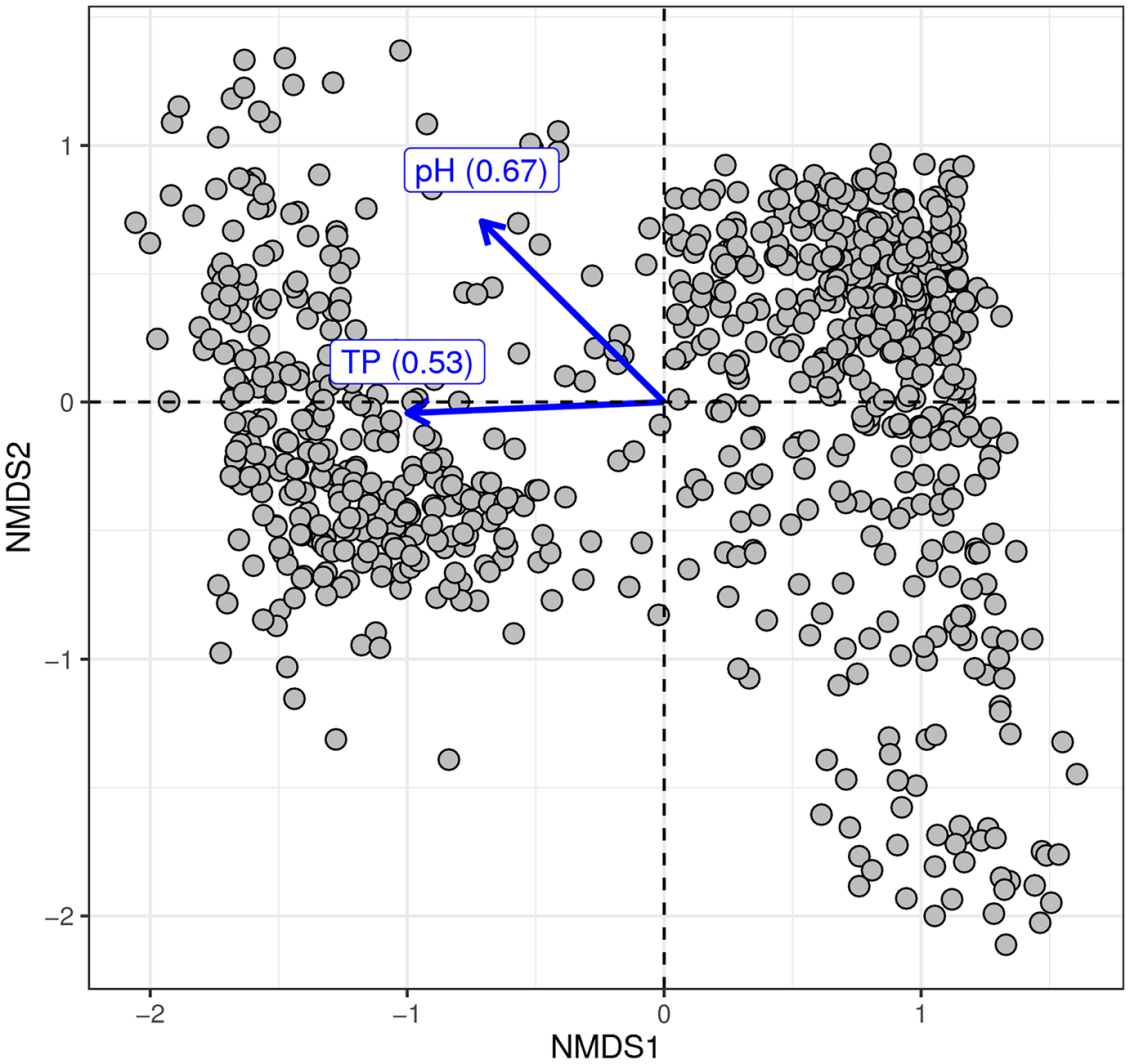
Similarity of diatom communities found in the 820 studied Swedish stream sites and their relation to total phosphorus and pH gradients (non-metric multidimensional scaling (NMDS) ordination plot of Bray–Curtis community dissimilarities based on diatom taxa’s relative abundances, with fitted gradients of TP and pH). Squared correlation coefficients are given for TP and pH reflecting the strength of those factors as a predictor of the assemblage structure. Stress value of NMDS ordination: 0.13

The PCA analysis showed that our sites were well aligned along a nutrient, and a secondary pH gradient (Fig. S2). The NMDS ordination analysis confirmed that the community structure of diatoms in Swedish streams was clearly related to both TP and pH (Fig. 2). While both factors were important, there was evidently a unique impact of each of them. Regarding TP, there was a gap between two clearly TP defined diatom communities (Fig. 2), but not between sites (Fig. S2).

With our study sites well spread along a TP gradient, and TP being important for diatom community structure, a meaningful TP index could be developed.

### Development of the Swedish phosphorus diatom index

**Fig. 3:**
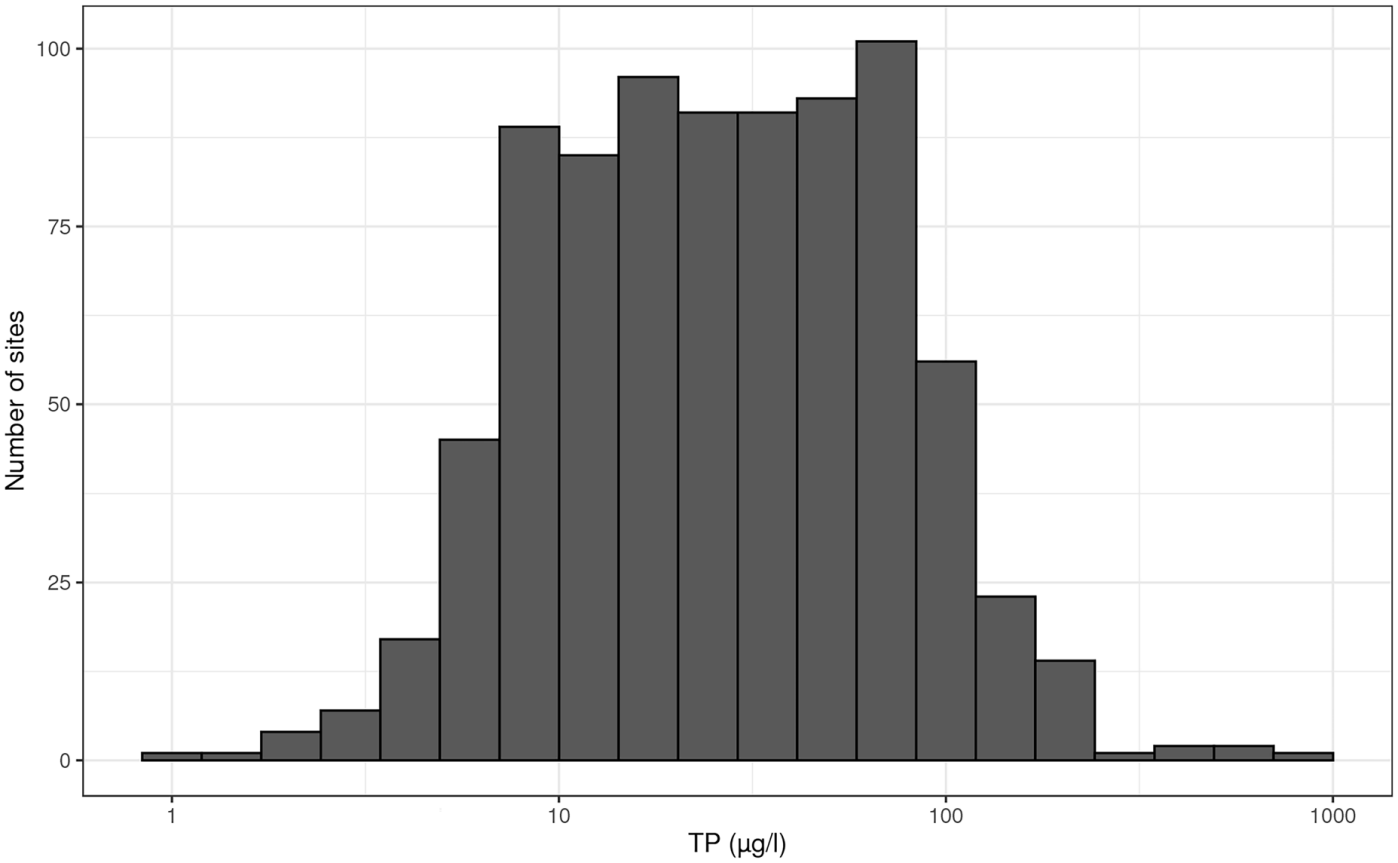
Measured TP concentrations (annual mean) of the studied sites (*n* = 820 Swedish streams)

**Fig. 4:**
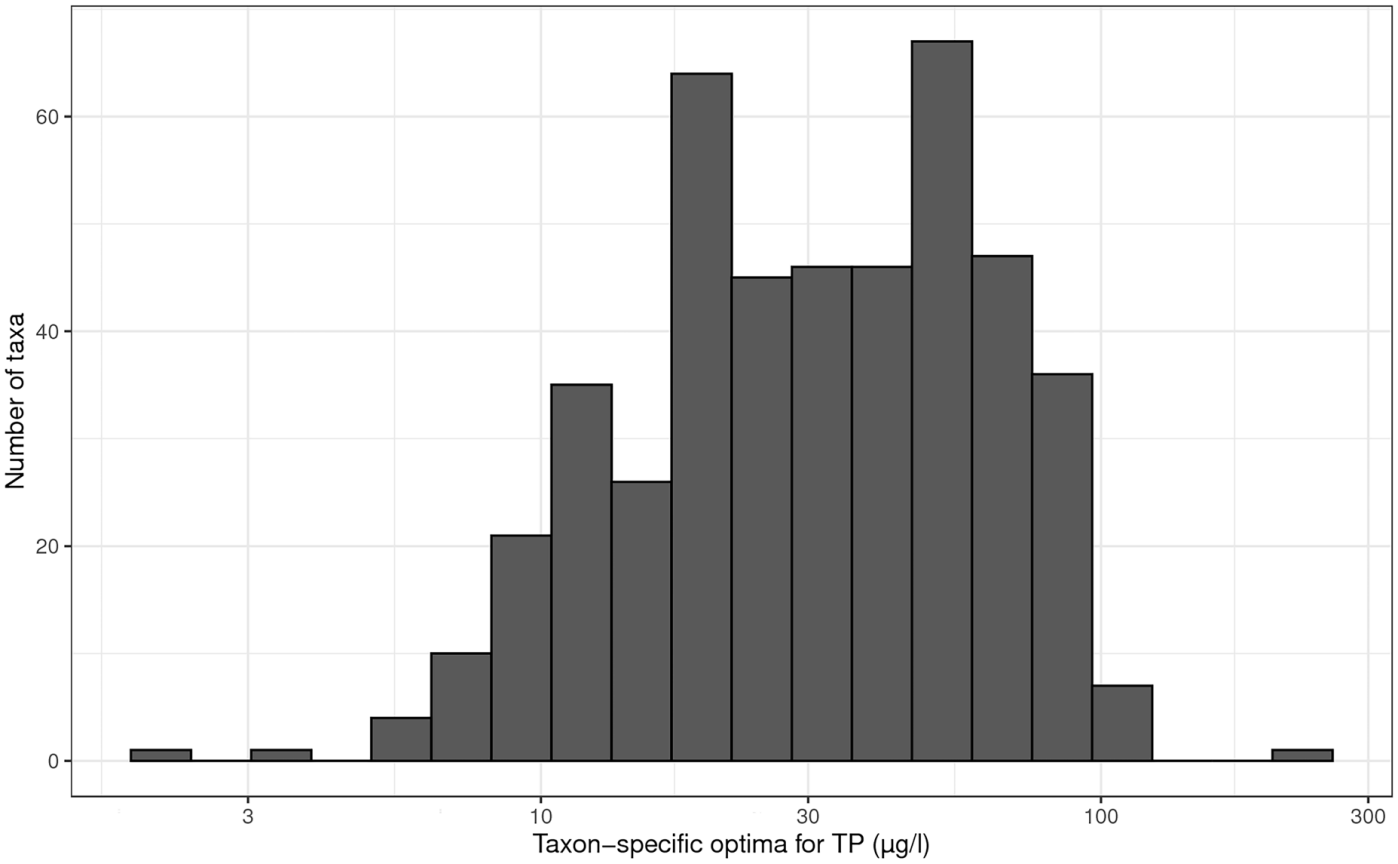
Taxon-specific TP optima obtained by modeling the abundance distribution of individual freshwater diatom taxa along the TP gradient and calculating the abundance-weighted average TP (*n* = 455)

**Fig. 5:**
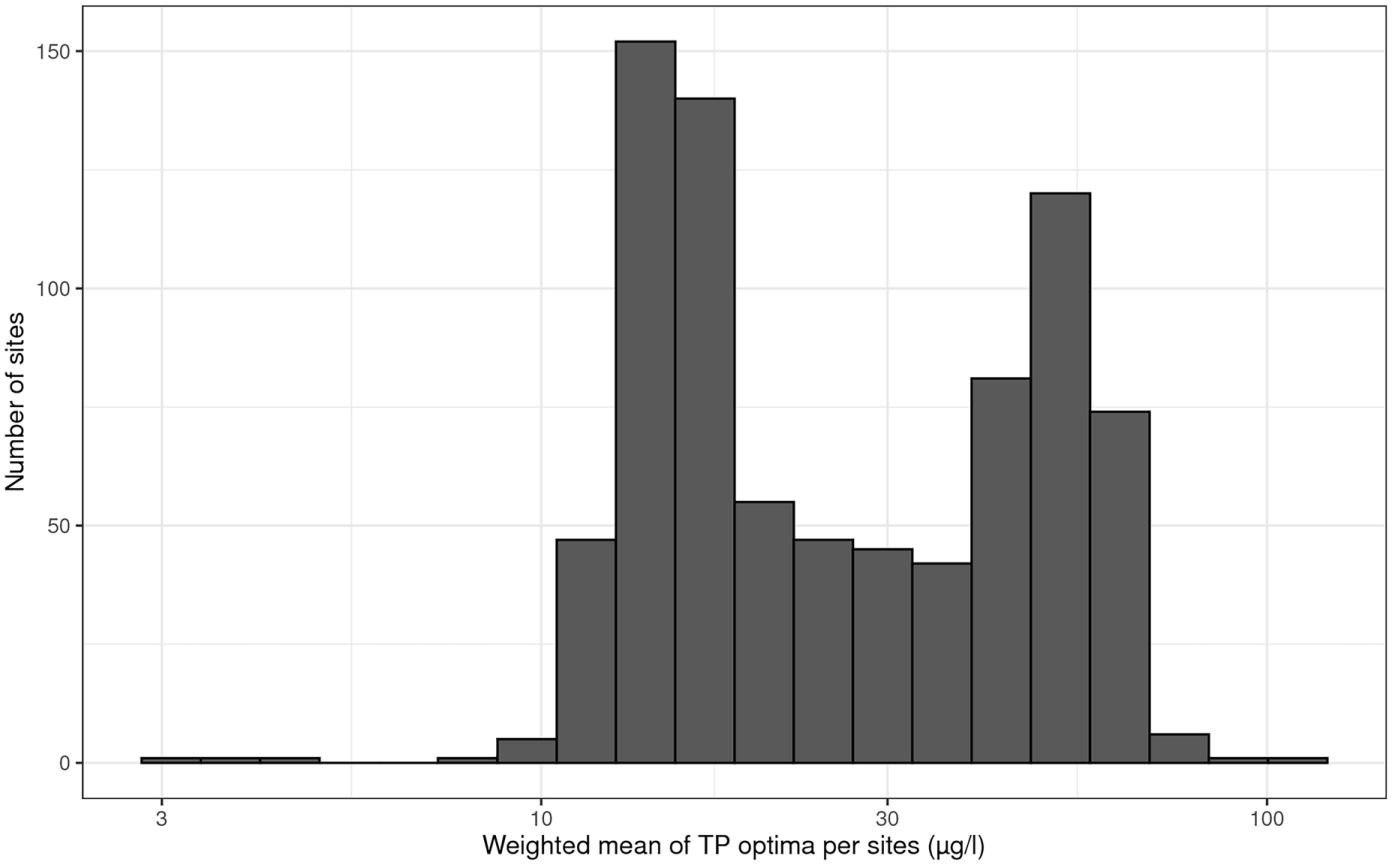
Site-specific TP optima of the studied sites (*n* = 820), calculated as abundance-weighted mean of the taxon-specific optima for each site

The TP range in our study was 1–902 (median 25, interquartile range 12–57) µg TP/l with a unimodal distribution (dip statistic *D* = 0.01, *p* = 0.69) (Fig. 3), thus sufficient to cover the known response range of Swedish benthic diatoms to TP (Kahlert & Gottschalk, 2014). The modeled taxa-specific TP optima covered the range from 2.2 to 238 µg TP/l and showed a unimodal distribution when log-transformed (dip statistic *D* = 0.02, *p* = 0.13). The interquartile range for the taxa-specific optima was 18–54 µg TP/l with a median of 31 µg TP/l (Fig. 4). In contrast, the site-specific optima showed bimodality (dip statistic *D* = 0.03, *p* < 0.01), with two peaks having a minimum of sites in between (Fig. 5). In other words, a site tended to have either a diatom community with a community average of a low TP optimum or vice versa of a high TP optimum. The number of sites having an average “lukewarm” medium TP optimum was few, showing a clear minimum in the range of 20–37 µg TP/l. This result was surprising, as both the distribution of the TP range and the taxa-specific optima were unimodal, but it reflected the observed gap of dissimilarities between diatom communities (Fig. 2). To smoothen this strong diatom response and enable a linear indication of TP, the PDI_SE_ was developed in a way to bridge the gap, namely we used a square root transformation of a diatoms relative abundance to relax the gap along the TP gradient.

**Fig. 6:**
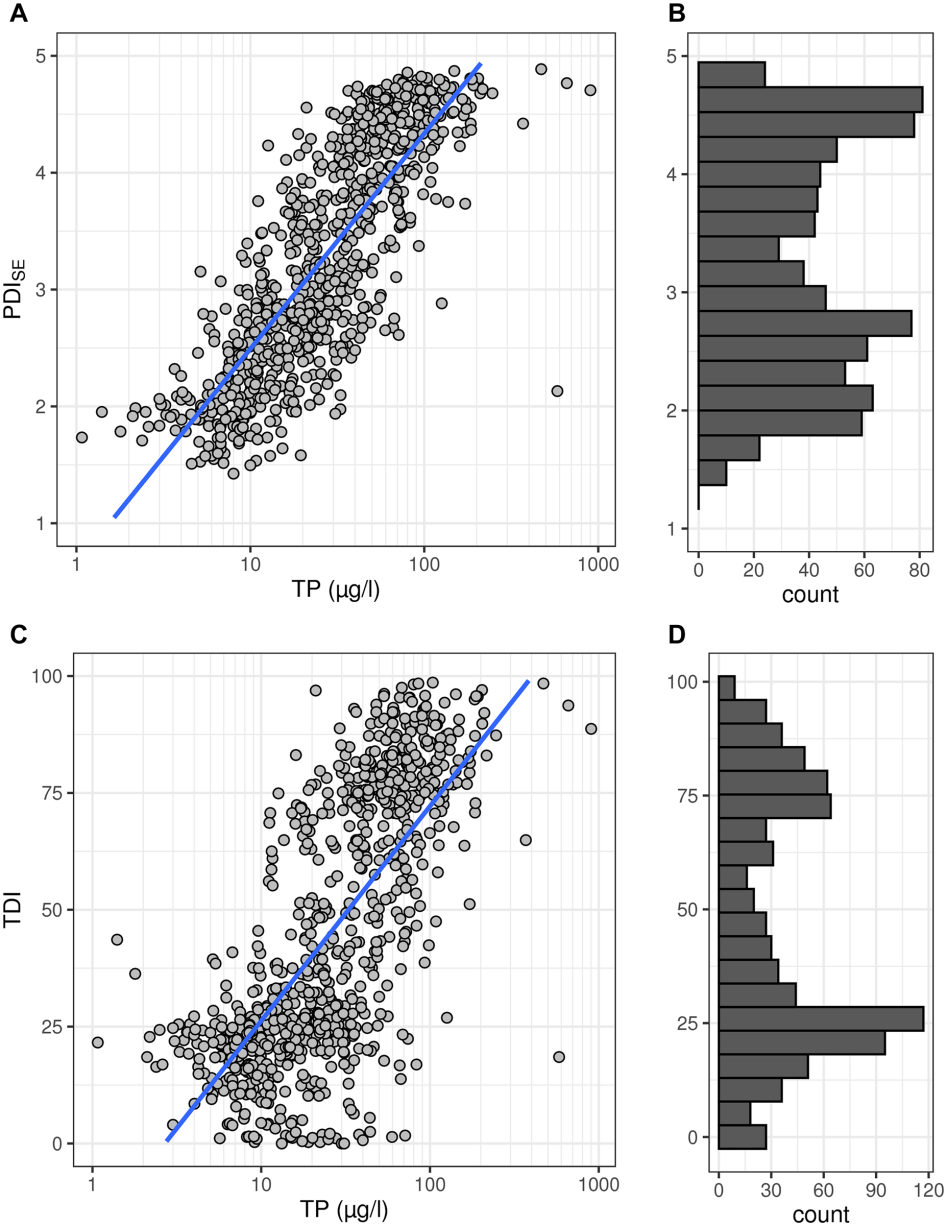
**A**  Correlation of PDI_SE_ with TP (PDI_SE_ = 1.843 * log_10_TP + 0.649; *r*^2^ = 0.68); **B** distribution of PDI_SE_ values (dip statistic *D* = 0.04, *p* < 0.01); **C**  correlation of TDI with TP (TDI = 45.92 * log_10_TP − 19.68; *r*.^2^ = 0.51); **D** distribution of TDI values (dip statistic *D* = 0.04, *p* < 0.01)

The final PDI_SE_ had a linear correlation with log_10_TP of *r*^2^ = 0.68 (Fig. 6). The outliers at very low and very high TP concentrations indicated that linearity was given for a range of about 4 to 100 µg TP/l. Before and after, the few existing data did not follow the linear model, but rather laid on a plateau. Our attempt of relaxing the gap between clustered diatom communities along the TP gradient was fruitful (Fig. 6B), even if it did not fully remove the bimodal distribution of PDI_SE_ values (dip statistic *D* = 0.04, *p* < 0.01).

### The bimodal pattern of response

**Fig. 7:**
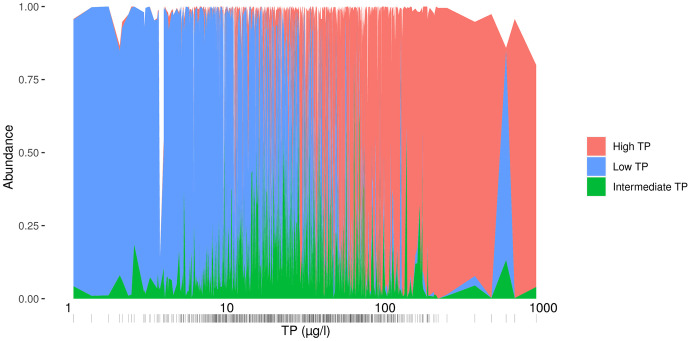
Relative abundance of diatom taxa grouped into three categories (low, medium, high TP preference) based on their TP optima (~ 0–20, 20–37, > 37 µg/l) along the measured site TP gradient (µg/l). Number of taxa in dataset of the respective taxa groups: low = 139, intermediate = 117, high = 199. Contribution to the relative abundance in a sample (average of their relative abundance among the 820 sites): low = 45%, intermediate = 15%, high = 37%

The bimodal pattern of the benthic diatom response assembling taxa in either a “low phosphorus” or a “high phosphorus” community, i.e., a diatom assemblage with an average of either low or high TP optimum (Figs. 2 and 5) has to our knowledge not been shown earlier. This pattern could not be explained by a gap of sites matching the minimum of the observed pattern (Fig. 3) nor by a matching pattern for the taxon-specific TP optima (Fig. 4). Our study shows that there was no lack of taxa with an intermediate TP optimum between 20 and 37 µg TP/l, even if their number (Fig. 4, 117 taxa) was a bit lower than of the taxa with low (*n* = 139) or high (*n* = 199) optimum respectively. However, we found that the intermediate taxa occurred with lower relative abundances even in the sites with intermediate TP concentrations (Fig. 7). While taxa with low or high TP optima had on average a relative abundance of 45% and 37% in a sample, respectively, the taxa with intermediate TP optima contributed on average only 15%. Thus, also sites with intermediate TP concentrations were dominated by taxa with low or high TP optima, and the taxa with intermediate TP optima did not form typical lukewarm communities.

Obviously, there are “lukewarm” diatom taxa preferring intermediate phosphorus concentrations; however, there seem to be no stable “lukewarm” diatom communities under natural conditions, at least in Nordic streams. It seems as if diatom assemblages with intermediate average community TP optima might be less stable, as there were much less sites with such assemblages. The unstable range in the middle of the studied TP gradient was found to cover the community optimum averages of about 20–37 µg TP/l. Earlier studies confirm that diatom and other algal assemblages are instable over the same TP range (Gottschalk, 2014 and references therein). However, these studies focused on the thresholds of measured TP concentrations where communities and taxa changed. Our study now analyzed the aggregated taxon composition, and the effects on the aggregated TP optima, over the range of measured TP.

The analysis of the diatom taxa composition of the sites with an average site-specific low, high, or intermediate TP optimum showed indeed that a typical intermediate assemblage seemed to be lacking. The SIMPER results confirmed that the taxa contributing most to group differences were associated with either the low TP or the high TP group, whereas the group of sites with intermediate average site-specific TP optima did not have a similar clear setup of distinct taxa, reflecting the gap between sites of similar composition shown earlier (Fig. 2 and Supplement Tab. S3). Eleven taxa contributed to a cumulative group difference of 50% between the low, intermediate, and high TP site groups (Supplement Tab. S3). The group of sites with high TP site-specific optima were characterized by a high relative abundance of *Achnanthidium minutissimum* (Kützing) Czarnecki group 3 (mean valve width > 2.8 µm) (contribution to cumulative group difference: 9.9%), *Cocconeis placentula* Ehrenberg incl. varieties (4.3%), *Amphora pediculus* (Kützing) Grunow (3.5%), and *Eolimna minima* (Grunow) Lange-Bertalot in Moser & al. (now *Sellaphora nigri* (De Not.) C.E. Wetzel et Ector comb. nov. emend.) (2.8%), whereas the low TP sites were characterized by *Achnanthidium minutissimum* (Kützing) Czarnecki group 2 (mean valve width 2–2.8 µm) (17.4%), *Fragilaria gracilis* Østrup (2.4%), *Eunotia incisa* Gregory (2.2%), *Tabellaria flocculosa* (Roth) Kützing (2.2%), *Brachysira neoexilis* Lange-Bertalot (2.1%), and *Eunotia minor* (Kützing) Grunow in Van Heurck (1.7%). Only the last of the taxa contributing to a cumulative difference of 50% between the low, intermediate, and high TP site groups was typical for the intermediate sites (*Staurosira venter* (Ehrenberg) Cleve & Moeller) (1.6%). *E. minor* was also a prominent example of the taxa with a rather “lukewarm” TP optimum (sensitivity class 3); however, it was equally abundant in both intermediate and low TP sites.

### PDI_SE_ has an improved response to TP compared to TDI

**Fig. 8:**
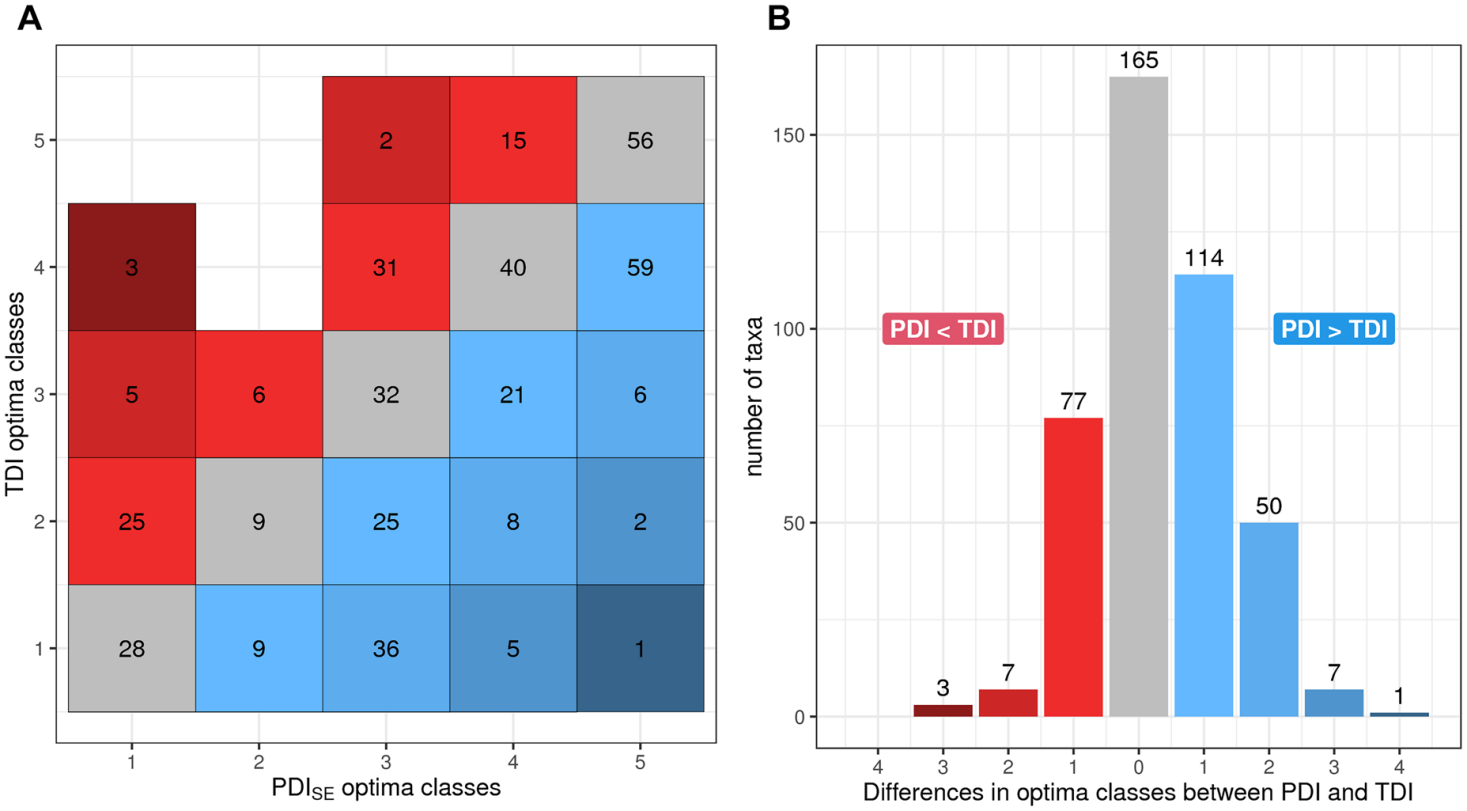
Comparison of the diatom taxon-specific sensitivity classes of the nutrient indices TDI and PDI_SE_. Both indices have five sensitivity classes, ranging from 1 = very sensitive to TP to 5 = indicating high TP. Sensitivity classes were more often similar between indices for high classes (4, 5) than lower classes (1, 2, 3). In general, more taxa were reclassified to a higher class for the PDI_SE_

The PDI_SE_’s response to TP was clearly improved compared to TDI currently used as supporting index in the Swedish standard method for diatoms (Fig. 6). The PDI_SE_ was higher correlated (*r*^2^ = 0.68) and had a more linear response than the TDI. Whereas the TDI also showed a clear response to TP with a correlation coefficient of *r*^2^ = 0.51, TDI values tended to cluster heavily at both ends of the TP range, with a gap in the middle with few values (Fig. 6C and D). One feature of the PDI_SE_ that handles this issue is the use of square root transformed abundance data. This transformation could reduce the effect of strong clustering at the two ends and promote a more homogenous distribution of PDI_SE_ values along the TP gradient and linear relationship. Another reason for the better correlation of the PDI_SE_ is the improved classification of the sensitivity value of the diatom taxa, adapted to Swedish conditions. The majority of the taxa changed class (Fig. 8), with a tendency to be categorized in a higher class, i.e., indicating higher P concentrations relatively to other taxa (Fig. 8). It is important to note that the reference gradient used to develop PDI_SE_ was total phosphorus, while filterable reactive phosphorus (FRP) was used to develop TDI. Although these two parameters usually correlate strongly, it may partly explain the better correlation of PDI_SE_ to TP. For example, we optimized the reflection of the lower part of the TP range by covering the TP range up to 32 µg TP/l with three sensitivity classes, while the TDI divides the lower FRP range up to 35 µg FRP/l into only two classes. While TP and FRP concentrations cannot be compared directly, the better coverage of the lower P concentrations could explain why some of the taxa changed one class up. However, several taxa changed more than one class up, and others changed classes down, i.e., showing a different realized niche than modeled in the UK for the original TDI development. Among the ten most frequent taxa which changed class, we found *E. minor* and *E. bilunaris* which both moved two classes up, from indicating low TP concentrations in the TDI to indicating intermediate TP concentrations in the PDI_SE_. On the other hand, *T. flocculosa* moved one class down, now indicating low TP concentrations in the PDI_SE_. We can only speculate about the causes. One possibility might be that the somewhat different geochemical background and climate might have impacted the response of a diatom to TP, another that many of the Swedish taxa might have been too rare in the UK database to model a robust TP optimum. It is also possible that the taxa were occurring in a somewhat different nutrient condition due to interactions, e.g., competition, with other diatom taxa, in constellations not occurring in Sweden. Furthermore, several taxa with the same name might actually be species complexes represented by different cryptic species in the two different countries. These cryptic species then might have different ecological optima in the two countries, as we have shown for example for *T. flocculosa* (Kahlert et al., 2021).

### Comparison of PDI_SE_ with IPS and TDI

**Fig. 9:**
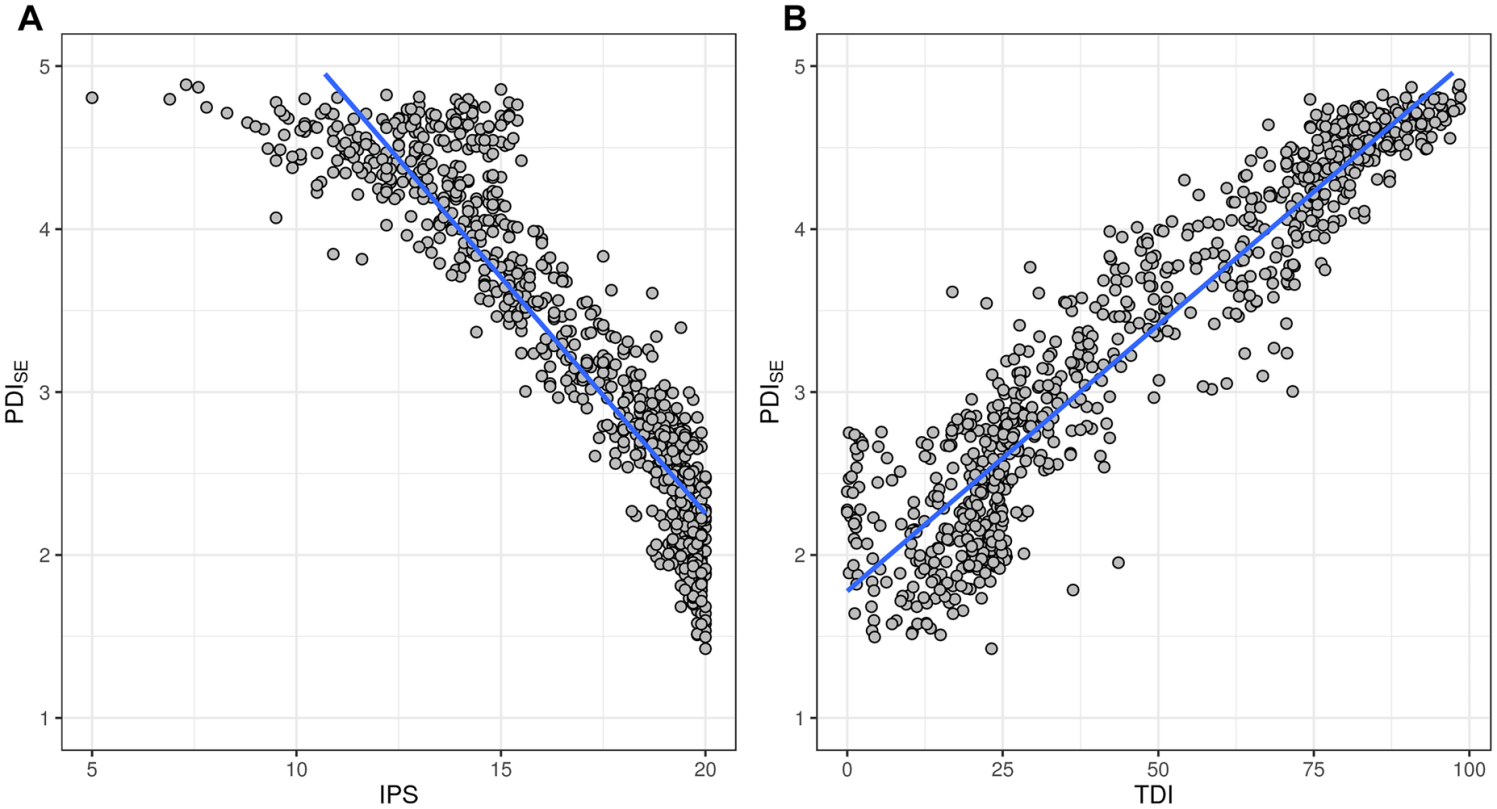
Correlation of PDI_SE_ with IPS (PDI_SE_ =  − 0.29 * IPS + 8.0536; *r*^2^ = 0.84) and TDI (PDI_SE_ = 0.03 * TDI + 1.7782; *r*.^2^ = 0.88)

The PDI_SE_ was well correlated to both Swedish standard indices IPS and TDI (Fig. 9A and B), indicating the possibility to implement the new index in the standard method, with the possibility to replace the TDI to guide the interpretation of the IPS as supporting nutrient index. The correlations of PDI_SE_ to both IPS and TDI were high on average; however, some sites were obviously classified differently with the PDI_SE_ compared to IPS and TDI (Fig. 9A and B). The correlation of the PDI_SE_ versus TDI and IPS was especially low for the nutrient poor sites, most probably because the PDI_SE_ classified those sites more correctly (Fig. 6).

### Comparison of the PDI_SE_ to other diatom nutrient indices

The PDI_SE_ is among the best nutrient indices developed for streams so far, with the correlation to TP (*r*^*2*^ = 0.68, Fig. 6) being among the highest reported for other benthic diatom nutrient indices globally (*r*^2^ = 0.01–0.79, Poikane et al., 2021). The correlation was better than for the current Swedish diatom indices IPS and TDI (Kahlert, 2011; Kahlert & Gottschalk, 2014; Kahlert et al., 2021; Fig. 6). Thus, the PDI_SE_ met the expectations for a robust nutrient index adjusted to Swedish conditions and reflects currently the optimum of phosphorus indication by freshwater benthic diatoms from environmental samples for Swedish streams. With that said, even the PDI_SE_ is not perfect, and one of the reasons is probably the bimodal pattern of response to phosphorus, which is a challenge when developing an index.

### Further improvements of the PDI_SE_

Still, many of the Swedish diatom taxa are for the moment not included in the calculation of the PDI_SE_ because their abundance was too low in the Swedish database to model an optimum. TDI on the other hand includes most of the taxa. The PDI_SE_ could potentially be improved by testing to use the existing TDI classifications for the missing taxa also for calculation of the PDI_SE_, and test if the outcome might get even more robust. Another further development of the PDI_SE_ would be to calibrate it for the use in lakes. Such an nutrient indicator would also in lakes complement the established and frequently used diatom method to assess ecological status (Kelly et al., 2014). We expect that benthic diatoms could be used to indicate lake TP concentrations because we have earlier shown that they are strongly related to TP in Swedish lakes (Kahlert & Gottschalk, 2014), and also respond earlier, and correlate better to nutrient concentrations than the currently mainly used organism group phytoplankton (Gottschalk, 2014). Similar results have been found by others as well (Rimet et al., 2015). Finally, we found that alpine streams, and eutrophicated streams with low pH, were underrepresented in our database. We recommend to test if the PDI_SE_ might need revision for different stream types, or lakes, as soon as sufficient data are available.

### Use of the PDI_SE_ in environmental assessment

The PDI_SE_ is no index to assess a general degradation of a stream. Instead, it is solely developed to reflect TP concentrations. Compared to a modeled reference concentration for phosphorus, the PDI_SE_ could however be used to assess a deviation from the reference status and indicate eutrophication. It could also be used to follow up countermeasures to minimize eutrophication. The PDI_SE_ will be especially valuable to indicate TP concentrations with less uncertainty than the TDI especially in oligotrophic sites, and upstream a lake to assess the risk for lake eutrophication.

Today, the Swedish benthic diatom standard is dedicated to assess ecological status as defined for the WFD (Havs- och vattenmyndigheten, 2018a), with a focus to indicate a general degradation of a water body, and to cover especially the good/moderate boundary where measures have to be taken to improve the ecological status class. With the aim to cover the entire degradation range from slight increases of nutrients to heavy organic pollution and oxygen depletion, this being harmonized for all European countries, some of which having large problems with organic pollution, the current diatom indices are not harmonized with, e.g., the plankton indices. Most of those have been explicitly developed to indicate eutrophication, the main cause for cyanobacterial blooms, in turn the main problem in many European lakes. The current diatom method is therefore not well adapted to indicate TP concentrations, or eutrophication alone, even if both IPS and TDI are correlated to TP. With the PDI_SE_, there now is a benthic diatom index which can be used to detect eutrophication in streams similarly to the planktonic indices in lakes. Furthermore, an adapted PDI_SE_ could be used in lakes as well for the assessment of TP. In this way, there now is the possibility to develop a better linkage between the recently updated chemical targets for environmental assessment with biological responses, and also to harmonize the diatom method to the method for plankton, avoiding sudden changes in ecological status class assessments just because of a change of the used biological quality element (Fölster et al., 2021).

Our new TP index now enabled us to calculate the TP threshold concentrations between the five different ecological status classes of the WFD ranging from high to bad ecological status in a more robust way than earlier, which is important for water authorities setting TP reduction targets. The new calculated TP threshold values using the PDI_SE_ were 18 µg/l for the high/good status boundary and 54 µg/l for the good/moderate boundary (Table 1). These results confirmed the TP values reported in the background report of implementation of the diatom method for Sweden (Kahlert et al., 2007). We have earlier found a change from a reference community to more tolerant communities in Swedish streams at a similar concentration (15 µg/l, Kahlert, 2014). We also found a sudden change of diatom guilds at 18 µg TP/l in Swedish lakes (Gottschalk, 2014). The threshold TP value of 54 mg/l then represents the boundary between the good and the moderate ecological status class. This important boundary had been defined as the crossover point where sensitive diatom species are replaced by species defined as tolerant to a general degradation of the habitat (Kahlert et al., 2007; Kelly et al., 2009). Note that the Swedish diatom method is not separating between stream types. A study on streams of the northeastern USA confirmed that a similar TP value (51 µg/l) implicated a threshold for a sudden switch from sensitive diatom taxa to tolerant ones (Smucker et al., 2013). The same study was also able to couple this change point to clear increase of the benthic chlorophyll concentration at about the same TP concentration (58 µg/l TP).

**Table 1:** Current Swedish standard IPS class boundaries, corresponding TP (total phosphorus) concentrations, corresponding PDI_SE_ values and from this derived corresponding TP concentrations. *EQR*, Ecological Quality Ratio (ecological status of a water body expressed as ratio between the measured quality of a biological parameter, and a reference value, following the European Water Framework Directive (WFD: European Parliament & Council of the European Union, 2000); *IPS*, Indice de Polluo-Sensibilité Spécifique (Cemagref, 1982); *PDI*_*SE*_, phosphorus diatom index of Sweden; *Na*, not applicable

Ecological class boundaries using the biological quality element freshwater benthic diatoms	EQR	IPS	TP range [µg/l]^1^	TP [µg/l] corresponding to boundary (derived from IPS)^2^	PDI_SE_^3^	TP [µg/l] corresponding to boundary (derived from PDI_SE_/IPS)^4^
Reference value	1	19.6	High: 10 (7.5–16)		2.37	9
High/good	0.89	17.5	Good: 31 (19–50)	~ 17.5	2.98	18
Good/moderate	0.74	14.5	Moderate: 76 (63–102)	~ 56.5	3.85	54
Moderate/poor	0.56	11	Poor: 102 (84–111)	No response to TP for > 100 µg/l	4.86	No response to TP for > 100 µg/l
Poor/bad	0.41	8	Bad: No data	na	5.73	na

^1^Median and interquartiles of TP [µg/l] for the five ecological status classes of the WFD (implementation of Swedish diatom method (Kahlert et al., 2007)

^2^Derived from the interquartiles (Kahlert et al., 2007)

^3^This study: PDI_SE_ =  − 0.29 * IPS + 8.0536

^4^This study: PDI_SE_ = 1.84322log_10_TP + 0.6493

## Conclusions

A new Swedish phosphorus diatom index (PDI_SE_) was developed. We recommend to replace the current supporting nutrient index TDI in the Swedish standard method with the PDI_SE_. The latter indicated TP concentrations with less uncertainty than the TDI, especially in oligotrophic sites. This better correlation was mainly caused by our reclassification of the TP optimum of many taxa. The linear response to TP was given in a range of about 4 to about 100 µg TP/l. One challenge for the development of the PDI_SE_ was the bimodal response of the diatom community to TP. We found our sites to be clustered into basically two diatom communities along the TP gradient. These two assemblages then had site-specific TP optima averaged from taxon-specific optima which indicated either low or high phosphorus levels. This bimodal response was not caused by a lack of sites with intermediate TP concentrations. Instead, it seemed that there was no typical community for the sites with an intermediate TP optimum.

## Supplementary Information

ESM 1 Detailed description of the method to selecting groups of sites with a community optimum of low, intermediate (“lukewarm”), and high TP site-specific optima. In order to define the thresholds, first a density plot based on kernel density estimation (Silverman, 1981) was created from the distribution data of the weighted means of TP optima per sites, and the location of the two peaks (modes) in log10 µg/l TP of the bimodal distribution were defined (Fig. S1A). We hypothesized an overlying combination of two symmetric unimodal distributions. In a first step, the range between the minimum log10 µg/l TP value to the log10 µg/l TP value of the first mode was defined and the first interquartile was selected (Fig. S1B). The difference between this point and the value of the first mode was added to the value of the first mode’s value, representing the threshold between the low TP and the “lukewarm” groups (1.30 log10 µg/l = 20.1 µg/l; Fig. S1C). Following this method, the range between the second mode and the maximum log10 µg/l TP value was defined, and the third interquartile was selected (Fig. S1B). The difference between this point and the value of the second mode was subtracted from the second mode’s value, representing the threshold between the “lukewarm” and the high TP groups (1.57 log10 µg/l =37.2 µg/l; Fig. S1C-D).(PNG 2483 kb)

High resolution image (TIFF 297 kb)

ESM 2 PCA analysis of the sampling sites clustered by their environmental variables: total phosphorus (TP), phosphate (P-PO_4_^3-^), pH, nitrate (N-NO_3_^-^), and ammonium (N-NH_4_^+^) (n: 513 sites with a full set of variables). The first two axes, indicating two important gradients (nutrients and pH), explained 83% of the variation between sites.(JPG 468 kb)

ESM 3 Details of the stream characteristics of the 820 study sites. Stream type classification according to the official water management administration system (Havs- och vattenmyndigheten 2017). Calculation of median, interquartile ranges and ranges for the chemical variables included.(XLSX 183 kb)

ESM 4 List of taxa included in the PDI_SE_ and their modeled TP optima and tolerance, together with sensitivity and indicator classes. Taxa are abbreviated with OMNIDIA diatom codes, find the full taxa name in the taxon list of the freshwater diatoms of Sweden (Kahlert et al., 2018). The OMNIDIA codes were originally created by Michael Coste, Director of Research at IRSTEA Bordeaux, France (Lecointe et al., 1993).(CSV 36.3 kb)

ESM 5 Simper (Similarity Percentage) results, showing which taxa are primarily responsible for the observed difference between three groups of sites with different site-specific Total Phosphorus (TP) optima (average of the abundance-weighted means of taxon-specific optima for TP based on the taxa present at the given site): low TP, intermediate and high TP optima sites. Taxa are sorted in descending order of contribution to group difference. The last three columns show the mean taxon relative abundance [%] in each of the three groups. Taxa are abbreviated with OMNIDIA diatom codes, find the full taxa name in the taxon list of the freshwater diatoms of Sweden (Kahlert et al., 2018). The OMNIDIA codes were originally created by Michael Coste, Director of Research at IRSTEA Bordeaux, France (Lecointe et al., 1993).(CSV 76.7 kb)

## Data Availability

The raw data analyzed during the current study are available from Miljödata-MVM. The generated data are included in this published article (and its supplementary information files). The full datasets generated or analyzed during this study are available from the corresponding author on reasonable request.
