# Supplementary figures and images for: No lukewarm diatom communities—the response of freshwater benthic diatoms to phosphorus in streams as basis for a new phosphorus diatom index (PDISE)
Source: Environ Monit Assess. 2023 Jun 6;195(7):807. doi: 10.1007/s10661-023-11378-4 (PMC10244268; doi:10.1007/s10661-023-11378-4)

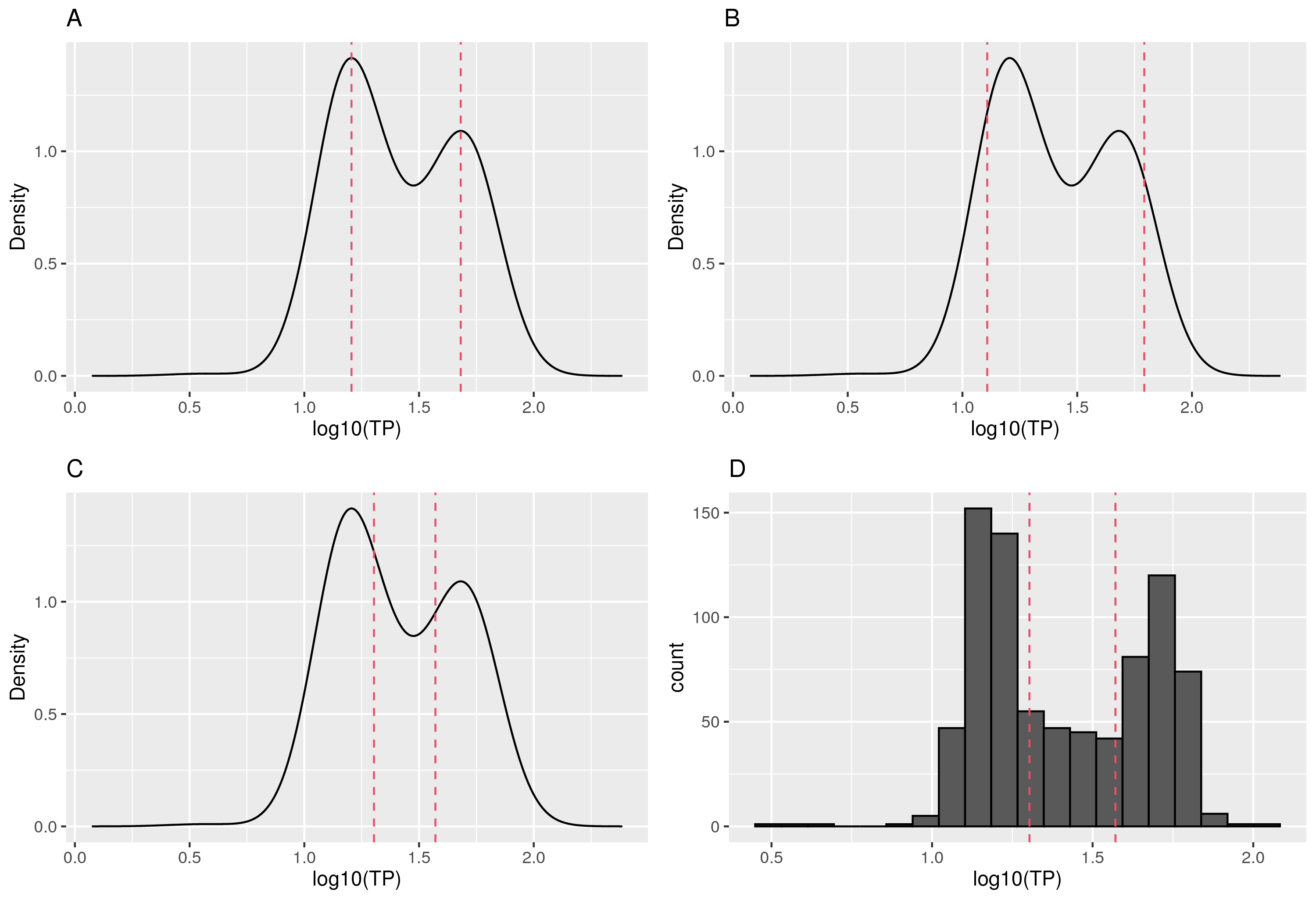

Supplement: Supplementary file 2 — High resolution image (TIFF 297 kb) [file 10661_2023_11378_MOESM1_ESM.tiff]

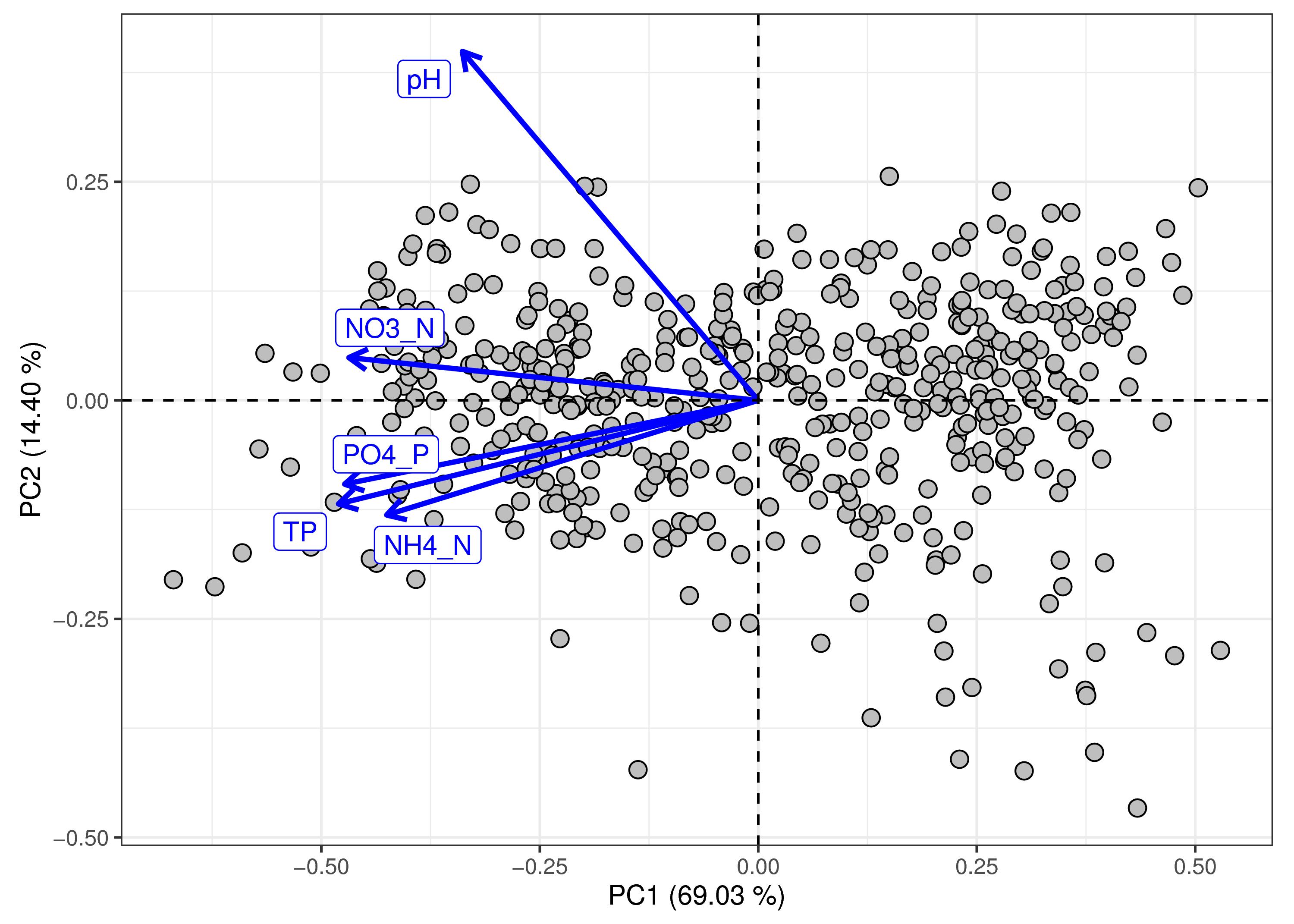

Supplement: Supplementary file 3 — PCA analysis of the sampling sites clustered by their environmental variables: total phosphorus (TP), phosphate (P-PO43-), pH, nitrate (N-NO3-), and ammonium (N-NH4+) (n: 513 sites with a full set of variables). The first two axes, indicating two important gradients (nutrients and pH), explained 83% of the variation between sites.(JPG 468 kb) [file 10661_2023_11378_MOESM2_ESM.jpg]
